# Fresh Versus Frozen Stool for Fecal Microbiota Transplantation—Assessment by Multimethod Approach Combining Culturing, Flow Cytometry, and Next-Generation Sequencing

**DOI:** 10.3389/fmicb.2022.872735

**Published:** 2022-07-01

**Authors:** Jaroslaw Bilinski, Mikolaj Dziurzynski, Pawel Grzesiowski, Edyta Podsiadly, Anna Stelmaszczyk-Emmel, Tomasz Dzieciatkowski, Karol Lis, Martyna Tyszka, Krzysztof Ozieranski, Łukasz Dziewit, Grzegorz W. Basak

**Affiliations:** ^1^Department of Hematology, Transplantation and Internal Medicine, Medical University of Warsaw, Warsaw, Poland; ^2^Department of Environmental Microbiology and Biotechnology, Faculty of Biology, Institute of Microbiology, University of Warsaw, Warsaw, Poland; ^3^Foundation for the Infection Prevention Institute, Warsaw, Poland; ^4^Department of Pharmaceutical Microbiology, Medical University of Warsaw, Warsaw, Poland; ^5^Department of Laboratory Diagnostics and Clinical Immunology of Developmental Age, Medical University of Warsaw, Warsaw, Poland; ^6^Department of Medical Microbiology, Medical University of Warsaw, Warsaw, Poland; ^7^First Department of Cardiology, Medical University of Warsaw, Warsaw, Poland

**Keywords:** fecal microbiota transplantation, conservation, gut microbiota, culturing, next-generation sequencing, flow cytometry, viability

## Abstract

The objective of this work was to compare the quality of FMT preparations made from fresh feces with those made from feces frozen at –30°C without any pre-processing or cryopreservation additives. The research hypothesis was that such preservation protocol (frozen whole stool, then thawed and processed) is equipotent to classical fresh FMT preparation. For that, three complementary methods were applied, including: (i) culturing in aerobic and anaerobic conditions, (ii) measuring viability by flow cytometry, and (iii) next-generation sequencing. Flow cytometry with cell staining showed that the applied freezing protocol causes significant changes in all of the observed bacterial fractions. Alive cell counts dropped four times, from around 70% to 15%, while the other two fractions, dead and unknown cell counts quadrupled and doubled, with the unknown fraction becoming the dominant one, with an average contribution of 57.47% per sample. It will be very interesting to uncover what this unknown fraction is (e.g., bacterial spores), as this may change our conclusions (if these are spores, the viability could be even higher after freezing). Freezing had a huge impact on the structure of cultivable bacterial communities. The biggest drop after freezing in the number of cultivable species was observed for Actinobacteria and Bacilli. In most cases, selected biodiversity indices were slightly lower for frozen samples. PCoA visualization built using weighted UniFrac index showed no donor-wise clusters, but a clear split between fresh and frozen samples. This split can be in part attributed to the changes in the relative abundance of Bacteroidales and Clostridiales orders. Our results clearly show that whole stool freezing without any cryoprotectants has a great impact on the cultivability and biodiversity of the bacterial community, and possibly also on the viability of bacterial cells.

## Introduction

Fecal microbiota transplantation (FMT) is a very effective treatment method in *Clostridioides difficile* infections (CDI) and other exploratory indications (Rokkas et al., 2019; DuPont et al., 2020; Ramai et al., 2021). To enable wide access to therapy for patients, various steps are taken to make FMT products commonly available. The most frequently used procedure is freezing the samples with the addition of cryoprotective agents, mostly glycerol.

Most studies applying frozen FMT have reported an overall CDI cure rate between 81 and 100% (Ramai et al., 2021). Two retrospective analyses (Hamilton et al., 2012; Satokari et al., 2015) and three randomized clinical trials (Youngster et al., 2014; Cammarota et al., 2015; Lee et al., 2016) directly compared fresh and frozen fecal preparations. All five studies reported no differences between fresh and frozen FMTs, despite the range in the storage time of frozen fecal matter (from 1 week to 6 months). Thus, current evidence indicates similar efficacy of frozen and fresh fecal preparations.

It is, however, postulated that glycerol can skew intestine microbiota composition and other natural methods should be applied (De Weirdt et al., 2010). Freezing the feces alone, without adding cryoprotectants, is the most common practice when collecting patient samples, but to the authors’ knowledge, it is also practiced in stool storage for the production of fecal microbiota preparations for transfer into the gastrointestinal tract of the recipient now, not only historically (Gustafsson et al., 1999) but also with very good results not differing from others (Grzesiowski et al., 2015). Different cryoprotectants have been described and tested, for example, freeze-drying FMT capsules (Staley et al., 2017; Burz et al., 2019). However, sole freezing in –20°C of the whole stool sample before preparation has not been described to date, although descriptions of such freezing may suggest that collecting and freezing all feces can be very effective (Vandeputte et al., 2017).

The objective of this work was to compare the quality of FMT preparations made from fresh feces with those made from frozen at –30°C without cryopreserving and processing before thawing. The research hypothesis was that such preservation protocol (freezed whole stool, then thawed and processed) is equipotent to classical fresh FMT preparation. For testing this hypothesis three complementary methods were applied, including: (i) culturing in aerobic and anaerobic conditions, (ii) measuring viability by flow cytometric method, and (iiii) next-generation sequencing (Bilinski et al., 2020). We postulated that the stool has protective properties for bacteria itself, and its processing before freezing is not obligatory, since anaerobic conditions inside the feces and low hydrated matter content may be a natural cryo- and viability-preservative.

## Materials and Methods

### Stool Donors, Stool Donations, and Processing Methodology

**FIGURE 1:**
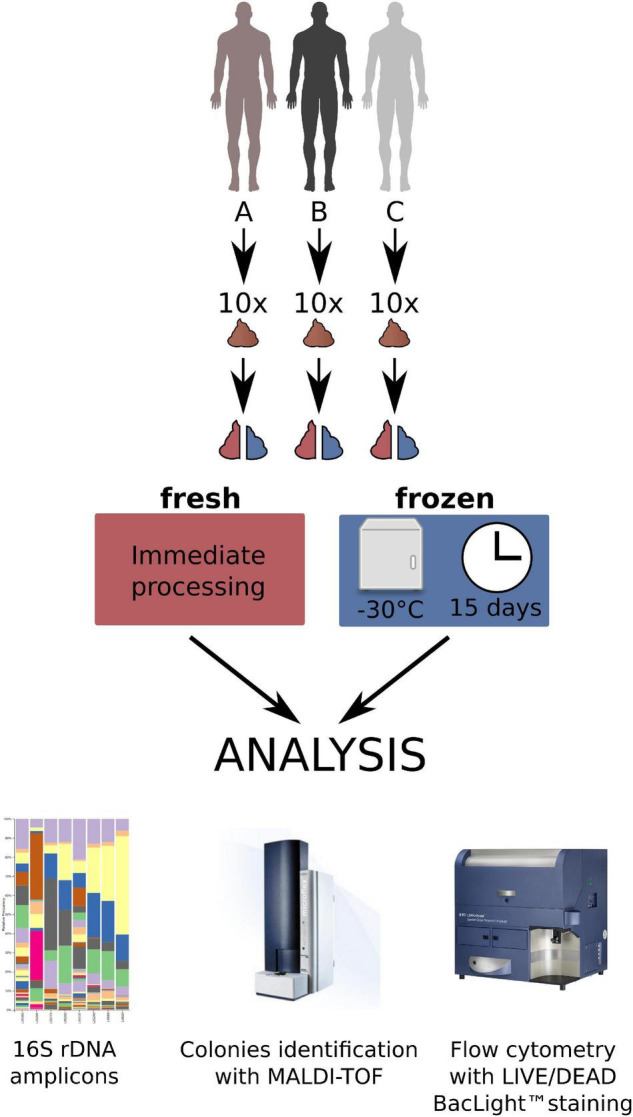
The research protocol of the study.

Ten consecutive stools donated by each of the three donors were used for the experiments (30 stools in total divided into two equal parts to prepare 60 fecal microbiota suspensions, 30 from fresh, and 30 from frozen stool). The donors were randomly selected (A and B) or intentionally chosen (C). In brief, one of them (donor C; male, 28 years old, healthy, with normal BMI) was a regular donor of feces to produce a preparation for the fecal microbiota transplantation (chosen from a stool donor bank) and the other two were randomly selected males (donor A—male, 16 years old with food allergy, recurrent aphthous stomatitis, and normal BMI; and donor B – male, 55 years old, with inhaled allergy, medical worker, with BMI 27). A medical questionnaire with basic data was received from each person. Each feces sample after donation and transportation to the laboratory was divided into two equal parts. Half of each stool was processed immediately and the second half was stored frozen, without any processing, at –30°C for a median of 15 days, and thereafter thawed and processed in the same way as the fresh stool sample. All samples were prepared in the same time frame and the same way in aerobic conditions by homogenizing, diluting in 0.9% NaCl, and sieving through sterile gauze or sieves to obtain a clear, homogeneous fluid being a suspension of feces. This is the regular way of producing feces for the use as FMT (Bilinski et al., 2017). The material both from fresh and frozen stool prepared in this way was then divided into three parts—one for assessment by the flow cytometry in the LIVE/DEAD method (Molecular Probes, Oregon, United States), one for performing classical culturing, and the third for immediate isolation of DNA for the V3-V4 16S rDNA variable regions sequencing (60 samples in total). Figure 1 shows the research protocol we used in this work.

### Flow Cytometry

Bacterial viability in fecal microbiota samples from fresh and frozen stool was measured by flow cytometry using LIVE/DEAD Bac*Light*™ Bacterial Viability and Counting Kit (L34856, Molecular Probes) according to manufacturer instructions (Molecular Probes, Oregon, United States). In brief, 977 μL of 0.9% NaCl, 1.5 μL of SYTO9, 1.5 μL of propidium iodide (PI), and 10 μL of the diluted sample were added to the flow cytometry analysis tube. Samples were 10-fold diluted in 0.9% NaCl. The tube was incubated for 15 min in the dark at room temperature and 10 μL of the microsphere suspension (beads) was added to the stained sample. The total volume of the sample in the flow cytometry analysis tube was 1000 μL. The samples were analyzed on an LSR Fortessa flow cytometer (Becton Dickinson, New Jersey, United States) with FACS Diva v8 software (Becton Dickinson). The gating strategy was as described in our first work (Bilinski et al., 2020). Three main cell populations were observed—alive, dead, and unknown (probably alive, probably dead) with a special not-alive-not-dead group of cells (SYTO9^–^PI^–^). The number of bacteria per mL in each analyzed gate was counted according to the following formula taken from the manufacturer materials:


$$\frac{\left(\left(\#ofevents\in gatedbacteriaregion\right)x\left(dillutionfactors\right)\right)}{\left[\left(\#ofevents\in beadregion\right)x10^{-6}\right]}=b\,a\,c\,t\,e\,r\,i\,a/m\,l$$

### Cultivation of Stool Microbiota

Samples of fecal microbiota suspension derived from each fresh and frozen stool were plated on six different agar media and incubated under conditions as follows: (i) CNA medium (colistin nalidixic acid agar; Oxoid, Basingstoke, United Kingdom)—for cultivation of Gram-positive aerobes, an enriched agar medium containing sheep’s blood, colistin, and nalidixic acid (to inhibit the growth of Gram-negative bacteria), and culture conditions: aerobic with 5% CO_2_, 37°C, 48 h; (ii) MacConkey medium (bioMérieux, Marcy l’Etoile, France)—for the isolation of Gram-negative rods, contains bile salts and crystal violet (to inhibit the growth of Gram-positive bacteria), and culture conditions: aerobic, 37°C, 48 h; (iii) Bile and esculin (CC) medium (Oxoid)—for the isolation and identification of bacteria belonging to the genus *Enterococcus*, which grow well in the presence of bile and have the ability to break down esculin, and culture conditions: aerobic, 37°C, 48 h; (iv) Schaedler Anaerobe KV Selective Agar with freeze-dried horse blood and the addition of kanamycin and vancomycin (bioMérieux)—a highly nutritious medium for the selective growth and isolation of anaerobic bacteria, especially of the genus *Bacteroides* and *Prevotella*, culture conditions: anaerobic, 37°C, 4 days; (v) Schaedler Anaerobe KV Selective Agar with freeze-dried horse blood (bioMérieux)—a highly nutritious medium for the isolation of absolute and relative anaerobes, culture conditions: anaerobic, 37°C, 4 days; (vi) Sabouraud agar with gentamicin and chloramphenicol (Oxoid)—selective medium for cultivation of mold and yeast, high glucose concentration, and presence of antibiotics (chloramphenicol and gentamicin) and acidic pH inhibits bacterial growth; the presence of antibiotics is another selection factor, and culture conditions: aerobic, 37°C, 10 days. The anaerobic incubations were carried out in anaerobic jars and atmosphere generators (Oxoid).

After the initial sample processing, colonies were selected (at least one colony per morphology) for identification using a Microflex LT mass and the MBT Compass IVD Biotyper software (Bruker Daltonics, Bremen, Germany). The colonies were deposited on a MALDI-TOF (Bruker Daltonics) target microflex and extracted with 5% formic acid, air-dried, and then overlaid with a 1-μL matrix solution of α-cyano-4-hydroxycinnamic acid in 50% acetonitrile and 2.5% trifluoroacetic acid. Two spots were examined for each colony. The Biotyper software was used to compare the protein profile of the cultured bacteria from a database of Bruker consisting of 2750 protein profiles. A score >1.9 was considered a reliable identification at the species level and a score >1.7 indicated the identification at the genus level. Strains of bacteria with scores lower than 1.7 were considered as unidentified.

To enumerate the number of colony-forming units (CFU) in the stool samples, 0.2 g of stool was diluted in 1 mL of phosphate-buffered saline (PBS), and 1 to 5 μL of diluted sample was spread on each media. Bacterial counts were recorded as CFU per gram of feces for each isolated species.

### DNA Sequencing

Total bacterial DNA was extracted using the Qiagen DNeasy Power Soil kit (Qiagen, Hilden, Germany) according to the manufacturer’s instructions and stored at –20°C. Using isolated DNA as a matrix, PCR reactions were performed in triplicate (to reduce PCR bias) using Bakt_341F 5′-CCTACGGGNGGCWGCAG-3′ and Bakt_805R 5′-GACTACHVGGGTATCTAATCC-3′ primer pair amplifying the variables V3 and V4 regions of the 16S rRNA genes (Herlemann et al., 2011; Klindworth et al., 2013). The electrophoretic analysis was performed for each of three replicates for the qualitative and quantitative evaluation of the PCR products. Then, products of three independent PCR reactions for each sample were mixed and used for the DNA sequencing as one amplicon to minimize the error due to the selectivity of the PCR reactions. The amplified PCR products were sequenced using the Illumina MiSeq instrument (Illumina, San Diego, CA, United States) in paired-end mode using a v3 chemistry kit (Illumina) at BIOBANK LAB (Chair and Department of Molecular Biophysics, University of Łódź, Poland).

### Bioinformatic Analysis

Sequencing data were processed with Qiime2 (version 2020.10) package (Bolyen et al., 2019). The reads were imported into Qiime2 and run through the dada2 plugin to obtain Amplicon Sequence Variants (ASV) (Callahan et al., 2016). Taxonomy was assigned for each of the ASVs using a pre-trained Naive Bayes classifier, based on Silva 132 99% database (Quast et al., 2013), which was trimmed to include only the V3 and V4 regions of 16S rRNA gene, bound by Bakt_341F and Bakt_805R primers sequences. Alfa and beta diversity metrics were generated using the following Qiime2 plugins: phylogeny (including mafft aligner and FastTree tool), diversity, and emperor (Price et al., 2010; Katoh and Standley, 2013).

### Statistical Analysis

Statistical differences between fresh and frozen samples were analyzed using Wilcoxon signed-rank test. To identify genera that differ in abundance between samples from different donors, ANCOM analysis was used (Mandal et al., 2015). All additional statistics and visualizations were generated using Python programming language with SciPy, matplotlib, pandas, numpy, and seaborn libraries (Hunter, 2007; Virtanen et al., 2020). Except when stated otherwise, p-values of less than 0.05 were considered statistically significant.

### Ethics

All subjects gave their informed consent for inclusion before they participated in the study. The investigations were carried out following the rules of the Declaration of Helsinki. According to local Bioethics Committee rules, for the non-intervention studies, no approval was needed to conduct this study.

## Results

### General Characteristics of Fresh Microbiota Samples

Results obtained for fresh samples have already been analyzed and described in our previous work (Bilinski et al., 2020). In brief, each method we applied (flow cytometry, classical culturing, and next-generation sequencing) has been shown to contribute to stool microbiota characterization differently, using a different perspective.

The flow cytometry analysis allowed for the evaluation of the total number of cells in each sample as well as live versus dead cell fractions analysis. In the performed analysis, a large fraction of cells not stained with one of the reagents, denoted Unknown, with an interesting subgroup, called SYTO9^–^PI^–^ fraction, composed of cells not stained by either of the reagents was revealed. Performed analysis showed that there were no statistically significant differences between cell numbers in fecal microbiota suspensions prepared from each donor stool and there were no significant differences in viability of cells for each donor. Noticeable domination of alive cells was observed.

The classical culturing to show whether this technique can reveal culturable bacterial indicators for “good” versus “bad” stool donors was applied. In total, 104 species representing 36 genera were identified. Classical culturing of fresh microbiota shows that donor C, being a regular stool donor, is characterized by the largest number of cultivable species (64) obtained from his stool versus other donors (48 species for donor A and 56 for donor B). Donor C’s stool had the largest number of unique species (29). The cultivable core microbiota, detected in the sample from all donors, was composed of only 16 species. In the next step, we evaluated the presence of identified species over time (throughout 10 sampling days) and we have shown that the plethora of bacterial species occurred only on individual days suggesting that single sampling can deliver non-representative and possibly false results. *Escherichia coli* was the only species detected in all samples.

An amplicon-based approach (i.e., metabarcoding combined with high-throughput taxonomical identification of bacteria) showed 97.75% of ASVs classified down to the genus level. The bacterial ASVs represented 18 classes, with Bacteroidia and Clostridia relative abundance constituting an average of 49.9% and 40.0%, respectively. At a genus level, the most dominant taxa were *Bacteroides* and *Faecalibacterium*, with relative abundance in each sample no less than 35% and 11%, respectively.

Alpha-diversity analysis showed that the Shannon index was similar for donors A and B, with its mean values equal to 10.11 and 10.02, respectively, while it was slightly, but significantly higher for the donor C–10.39 (*p* = 0.0191 for donor A vs. C and *p* = 0.0005 for donor B vs. C according to Kruskal–Wallis test, H value = 12.18).

ANCOM analysis, testing for taxa differences between donors, showed that when donors A and C were compared, the first could be characterized by *Anaeroplasmatales* and *Gastranaerophilales* orders, while the latter was characterized by an increase in abundance of *Acidaminococcus* and *Paraprevotella* genera. ANCOM analysis on donor B versus donor C pair showed an increase of *Anaeroplasma* and *Holdermanella* genera with Muribaculaceae and Puniceicoccaceae families defining donor B, and *Lachnospiraceae* and *Dialister* relative abundances significantly increased in donor C samples.

Pearson correlation coefficients between the double negative group of cells (SYTO9^–^PI^–^) and genera-level taxonomy data showed that the relative abundance of *Anaeroplasma* is positively correlated with the double negative group per sample percentage (ρ = 0.6312), followed by *Sanguibacteroides* (ρ = 0.4592).

Beta-diversity analysis on the Bray–Curtis Dissimilarity index showed that the overall internal similarity of time-resolved samples from donor C was much higher than for other donors. Clustering of the bacterial composition of feces in donor C indicated the most stable composition of intestinal microbiota over time.

### General Characteristics of Frozen Microbiota Samples

#### Flow Cytometry

**FIGURE 2:**
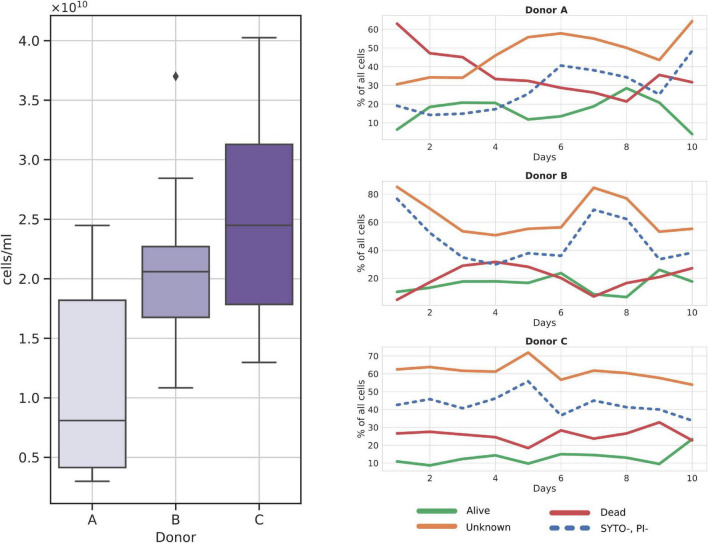
Cytometry cell count charts in frozen samples. **(Left panel)** Total cell counts per donor over all samples; **(Right panel)** relative changes in cell fractions throughout the sampling days. SYTO-, PI- fraction is additionally dashed as it is a subgroup of the Unknown fraction.

Frozen samples reported on average of 1.89*10^10^ cells per ml of prepared suspension, with donor C showing the highest average number of cells, 2.5*10^10^ cells/ml, followed by donor B, 2.09*10^10^ cells/ml, and donor A with 1.08*10^10^ cells/ml (Figure 2). Statistical testing with the Kruskal–Wallis test followed by the pairwise Dunn test showed a statistically significant difference between cell counts obtained from donor A and donor C samples (ρ = 0.0069). No other significant result was obtained when comparing the remaining pairs that were A versus B (ρ = 0.06) and B versus C (ρ = 0.36).

Among the three major cell groups, the Unknown group was usually the dominant one, with a mean sample contribution of 47.16%, 64.08%, and 61.16% for donors A, B, and C, respectively (Figure 2). Contrarily, alive and dead cells constituted comparably small percentages of cells across all samples, with a mean of 15.08% for alive and 27.45% for dead groups. Interestingly, SYTO9^–^PI^–^ (cells not stained with either dye) cells, a subgroup of the Unknown, nearly always constituted a regular percentage of the Unknown group, without major differences between donors. Furthermore, we noted that donor C samples could be characterized by their regular cell group distribution in every sample (Figure 2).

#### Classical Culturing

**FIGURE 3:**
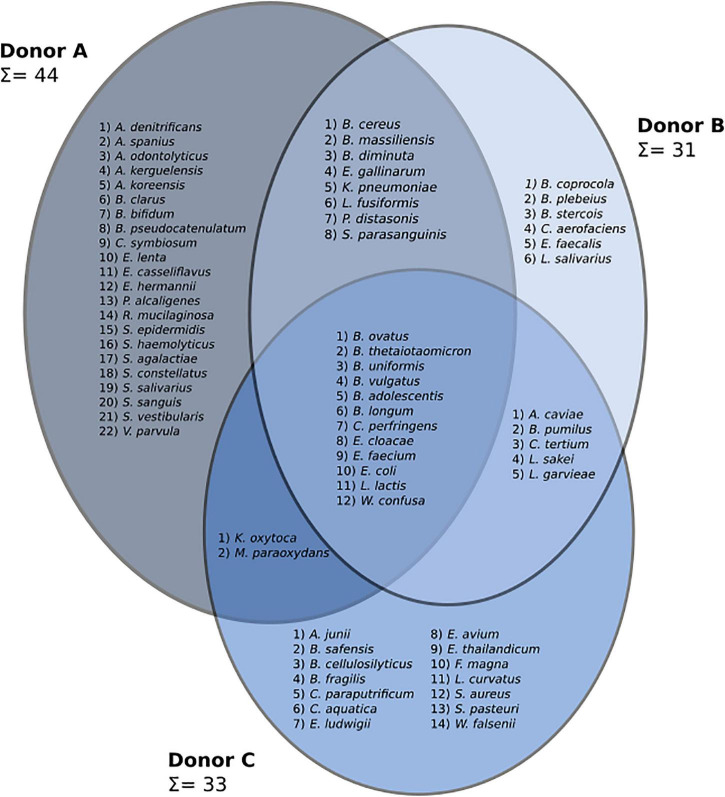
A three-set Venn diagram showing species discovered in frozen samples from each patient. Identified genera are as follows: *Achromobacter* (*A. denitrificans*, *A. spanius*), *Acinetobacter* (*A. junii*), *Actinomyces* (*A. odontolyticus*), *Aeromonas* (*A. caviae*), *Arthrobacter* (*A. kerguelensis*, *A. koreensis*), *Bacillus* (*B. cereus*, *B. pumilus*, *B. safensis*), *Bacteroides* (*B. cellulosilyticus*, *B. clarus*, *B. coprocola*, *B. fragilis*, *B. massiliensis*, *B. ovatus*, *B. plebeius*, *B. stercois*, *B. thetaiotaomicron*, *B. uniformis*, *B. vulgatus*), *Bifidobacterium* (*B. adolescentis*, *B. bifidum*, *B. longum*, *B. pseudocatenulatum*), *Brevundimonas* (*B. diminuta*), *Clostridium* (*C. paraputrificum*, *C. perfringens*, *C. symbiosum*, *C. tertium*), *Collinsella* (*C. aerofaciens*), *Comamonas* (*C. aquatica*), *Eggerthella* (*E. lenta*), *Enterobacter* (*E. cloacae*, *E. ludwigii*), *Enterococcus* (*E. avium*, *E. casseliflavus*, *E. faecalis*, *E. faecium*, *E. gallinarum*, *E. thailandicum*), *Escherichia* (*E. coli*, *E. hermannii*), *Finegoldia* (*F. magna*), *Klebsiella* (*K. oxytoca*, *K. pneumoniae*), *Lactobacillus* (*L. curvatus*, *L. sakei*, *L. salivarius*), *Lactococcus* (*L. garvieae*, *L. lactis*), *Lysinibacillus* (*L. fusiformis*), *Microbacterium* (*M. paraoxydans*), *Parabacteroides* (*P. distasonis*), *Pseudomonas* (*P. alcaligenes*), *Rothia* (*R. mucilaginosa*), *Staphylococcus* (*S. aureus*, *S. epidermidis*, *S. haemolyticus*, *S. pasteuri*), *Streptococcus* (*S. agalactiae*, *S. constellatus*, *S. parasanguinis*, *S. salivarius*, *S. sanguinis*, *S. vestibularis*), *Veillonella* (*V. parvula*), *Wautersiella* (*W. falsenii*), and *Weissella* (*W. confusa).*

A three-way Venn diagram was prepared to show bacterial species detected in frozen samples from each donor (Figure 3). In total, 69 species representing four phyla (*Actinobacteria*, *Bacteroidetes*, *Firmicutes*, and *Proteobacteria*) were found. Culturing experiments detected 44, 31, and 33 bacterial species in samples from donors A, B, and C, respectively. Core microbiota, common to all donors, was made of 12 different species. Frozen samples from donor A contained the highest number of unique bacterial species (22), while the unique number of species in donors B and C were 6 and 14, respectively. Samples from donor C reported the highest species persistence, which is the number of species detected in all samples from a given donor. There were four species detected in all samples from donor C (*Bacteroides vulgatus*, *Escherichia coli*, *Lactococcus garvieae*, and *Weissella confusa*) followed by donor B with three such strains *(Bacteroides ovatus*, *Escherichia coli*, and *Lactococcus garvieae*) and donor A with only *Escherichia coli* detected in all samples.

Isolated strains were classified as either aerobic or anaerobic based on their assigned taxonomy. The highest number of unique aerobic and anaerobic species was recovered for donor A, 27 aerobic and 17 anaerobic, followed by donor C, 20 aerobic and 13 anaerobic, and donor B with 16 aerobic and 15 anaerobic strains. Statistical analysis showed significant differences in the number of recovered aerobes between donors B and C (ρ = 0.037).

#### Next-Generation Sequencing

A total of 5,974,017 reads were obtained from Illumina MiSeq sequencing, with reads per sample ranging from 107,041 to 264,007. Quality control and merging of paired-ended reads using the *dada2* software package, resulted in the retention of, on average, 88,513.23 paired reads (SD = 16,406.09) per sample (Supplementary Table 1). Both the Nonpareil 3 and alpha rarefaction analysis (Qiime2 *diversity* plugin) showed sequencing depth close to 100%. Overall, 11,131 different ASVs were discovered, with feature frequency ranging from 1 to 10,539 merged sequences.

**FIGURE 4:**
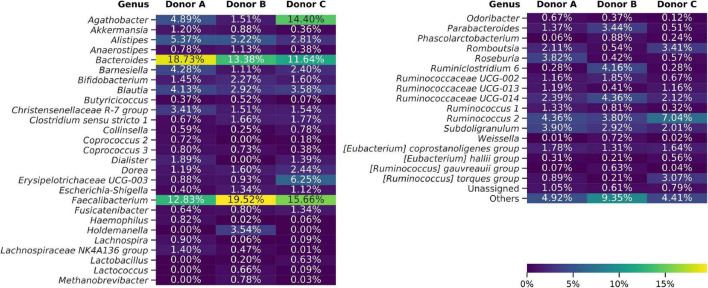
Heat map showing bacterial genera detected using amplicon sequencing (V3–V4 region of 16S rDNA). Averaged data for each donor are presented. The “Others” group summarizes genera with individual abundances lower than 0.5% in each sample. Sequences unassigned at the genus level were grouped and named “Unassigned.”

Taxonomy for each ASV was assigned using the Naive Bayes Classifier, trained on the Silva 132 database, and showed 93.38% of ASVs classified down to the genus level. Overall classification showed that 99.49% of all ASVs were bacterial, 0.34% archeal, and less than 0.17% were unclassified. The bacterial ASVs represent 18 classes, with Bacteroidia and Clostridia relative abundance constituting on average of 24.32% and 62.38%, respectively. Frozen samples were dominated by *Faecalibacterium* (4.56%–31.42%), *Bacteroides* (0.98%–26.98%), *Agathobacter* (0.18%–25.74%), and *Ruminococcus* 2 (0.55%–24.78%) genera (Figure 4). Differential analysis of frozen samples composition between donors highlighted several statistically different genera. ANCOM analysis showed that *Dialister* and *Coprococcus 2* genera were reported more frequently in samples from donors A and C than B. *Lachnospiraceae* NK4A136 group was more abundant in samples from donors A and B. Samples from donors B and C, in contrast to samples from donor A, reported an increased relative abundance of *Paraprevotella* genus. *Holdemanella*, *Methanobrevibacter*, and *Ruminococcaceae* UCG-004 were unique to samples from donor B, while *Peptococcus* was regularly detected only in samples from donor C.

**FIGURE 5:**
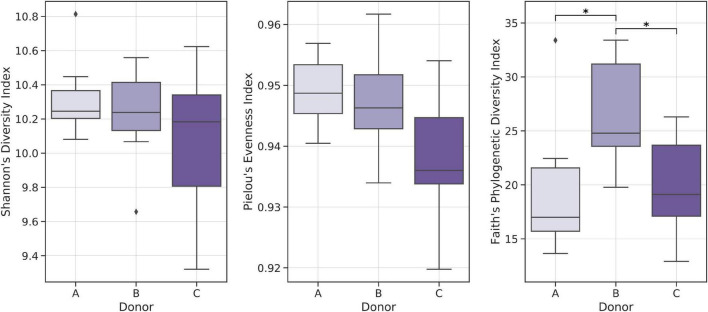
Boxplots showing distributions of selected biodiversity indices. Kruskal–Wallis test was used to detect statistically significant differences. *p < 0.01.

Alpha-diversity indices analysis showed relatively lower Shannon Diversity and Pielou’s Evenness indices in samples from donor C when compared with other frozen samples; however, no statistically significant result was obtained. Comparative analysis of Faith’s Phylogenetic Diversity (PD) between donors showed that frozen samples from donor B had the highest average Faith’s PD, with medians equal to 17.00, 24.79, and 19.12 for donors A, B, and C, respectively (Figure 5).

Beta-diversity analysis using classical indices, such as Bray–Curtis dissimilarity index, Jaccard index, and unweighted Unifrac, showed clear clusters, one per donor. However, the clusters were much less visible on PCoAs plotted using the weighted Unifrac distance metric, which also takes relative abundances into account (Supplementary Figure 1). This shows that each donor has his unique microbiota species, but they are not the dominant ones, as incorporating relative abundances blends all samples into one big cluster. This cluster is further stretched along one axis, which could mean that there is an important gradient in the relative abundance of one or more ASVs in all samples.

### Comparative Analysis of Fresh and Frozen Fecal Microbiota Suspensions

#### Flow Cytometry

**FIGURE 6:**
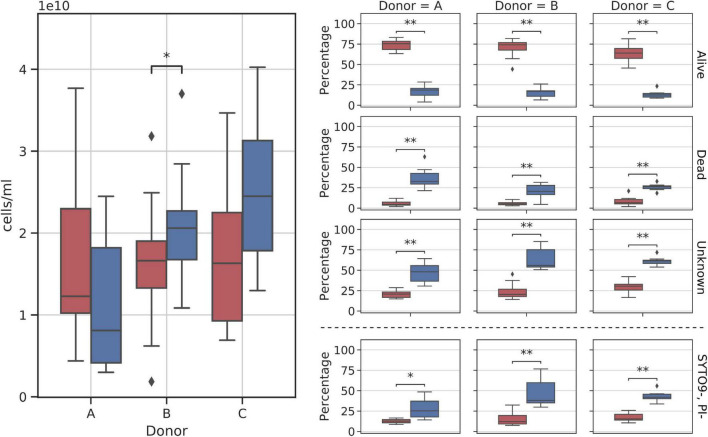
Diagram comparing flow cytometry cell counts and group fractions obtained for fresh and frozen suspensions. The **(left panel)** shows the total donor cell counts comparison. The **(right panel)** shows changes in each of the inspected flow cytometry groups (alive, dead, unknown, and SYTO9^–^, PI^–^ subgroups). Red—fresh samples; blue—frozen samples; * – *p* < 0.05; ** – *p* < 0.01.

Cytometry reported an average of 2.27*10^10^ cells across all samples (median = 2.21*10^10^). When comparing fresh with frozen samples, only one pair, donor B samples, showed slightly but significantly higher cell counts in frozen suspensions when compared with the fresh ones (mean fresh cell count: 1.63*10^11^, mean frozen cell count: 2.08*10^11^) (Figure 6).

Stool suspension cell counts were divided into three main groups: alive, dead, and unknown (not stained with either one of the reagents). Alive or dead groups were named so, if there was no doubt that they were clustered as alive or dead. In addition, we decided to highlight one more group denoted as SYTO9^–^PI^–^. It is a subgroup of “unknown,” not stained by both reagents considered as “double negative” (we think this may be a bacterial spores fraction or bacterial cells fraction with a particularly thick cell wall). A clear change in cell counts in each of those groups was detected as a result of whole stool freezing (Figure 6). Freezing and thawing the whole stool to prepare fecal microbiota suspensions resulted in a nearly 4-fold reduction of alive cell counts in samples from all donors. On average, 68.88% (SD = 10.85%) of cells in each fresh sample were categorized as alive, while alive cells constituted only 15.08% (SD = 6.08%) of all cells in frozen samples. The percentage of dead cells increased on average four times (fresh: 6.51%, frozen: 27.45%), while the unknown group grew by a factor of two (fresh: 24.60%, frozen: 57.47%). The SYTO9^–^PI^–^ subgroup reported 2.5-fold increase (fresh: 15.16%, frozen: 39.20%). All changes between fresh and frozen groups were statistically significant.

Samples from donor C appeared to react more consistently to freezing than samples from the other two donors. Figure 6 shows that cell count distributions of frozen samples from donors A and B show much more variability than those from donor C. This could be explained by the more stable composition of donors’ C microbiota over the sampling time, as was proved previously (Bilinski et al., 2020).

#### Classical Culturing

**FIGURE 7:**
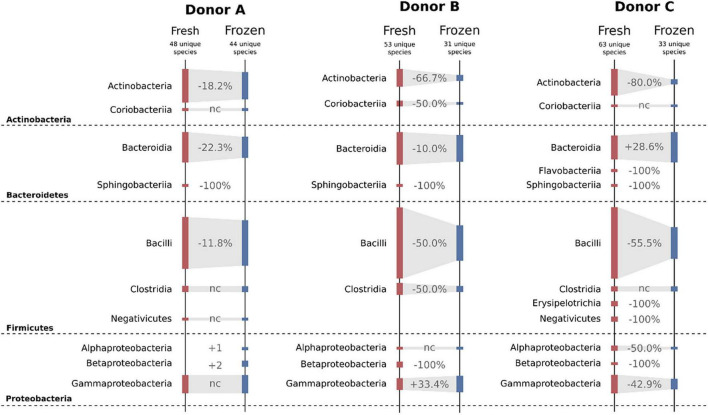
Diagram showing changes in cultivable bacteria between suspensions prepared from fresh and frozen stools. Dashed horizontal lines separate bacterial phyla. Values between bars show changes in the number of detected species. nc – no changes.

Freezing had a huge impact on the structure of cultivable bacterial communities. Overall, four different bacterial phyla were detected in samples from each donor. Variability between donors and their fresh and frozen samples is shown in Figure 7. When inspected on the class taxonomic level, the biggest drop in the number of cultivable species was observed for Actinobacteria and Bacilli. Donor C, a regular stool donor, reported the highest number of classes in fresh samples—12 classes. Five of those 12 classes were undetectable in suspensions from frozen samples, which were Betaproteobacteria, Erysipelotrichia, Flavobacteriia, Negativicutes, and Sphingobacteriia. Inspection of donor C’s unique species, detected in both fresh and frozen samples yielded only three species: *Bacteroides cellulosilyticus*, *Enterococcus avium*, and *Enterococcus thailandicus.* Interestingly, suspensions prepared from donor A frozen samples showed two bacterial classes undetected in his fresh samples, which were Alphaproteobacteria and Betaproteobacteria.

Experiments on fresh samples detected 103 bacterial species, while after freezing, the same samples delivered only 69 species; 47 of those 69 species were also detected in the fresh samples, while 22 were unique to frozen samples. Donor C was characterized by the biggest drop in the number of detectable cultivable species, a drop from 63 to 33, followed by donor B, 53 to 31, and donor A, 48 to 44 (Figure 7). However, we observed very high variability on lower taxonomic levels such as genus and family levels. In a few cases, there were species-level shifts between fresh and frozen samples, for example, in donor A, *Clostridium tertium* present in fresh samples was not detectable in frozen samples, while frozen samples reported previously undetected *Clostridium perfringens*. In addition, it is important to notice that only 31 of 103 detected bacterial species were found in suspensions prepared from the same stool before and after freezing. This further supports our conclusion from the previous work, that although the MALDI-TOF method is very precise, it requires repetitive sampling overtime when exploring diverse bacterial communities. Interestingly, frozen samples have shown a higher number of “highly persistent” species, that is species detected in all samples from the same donor. In the fresh samples, only *Escherichia coli* has been detected in all samples, while in the frozen samples that was valid for *Bacteroides ovatus*, *Bacteroides vulgatus*, *Escherichia coli*, *Lactococcus garvieae*, and *Weissella confusa*.

#### Next-Generation Sequencing

A total of 60 amplicon libraries, 30 prepared from fresh and 30 from frozen suspensions, was included in this analysis. As all libraries were prepared simultaneously, there were no significant differences between the number of obtained sequencing reads between fresh and frozen samples. For the needs of comparative analysis, 49,706 sequences were sampled from each sample. Alpha-rarefaction analysis with observed ASV counts and Good’s coverage showed that obtained subsamples accurately represented their original counterparts.

Alpha-diversity analysis was conducted using the Shannon Diversity index, Pielou’s Evenness index, and Faith’s Phylogenetic Diversity index. In most cases, selected biodiversity indices were slightly lower for frozen samples, but no statistically significant differences were detected when comparing fresh and frozen samples from the same donor. There were also no significant differences in comparisons of selected biodiversity indices between all fresh and all frozen samples.

**FIGURE 8:**
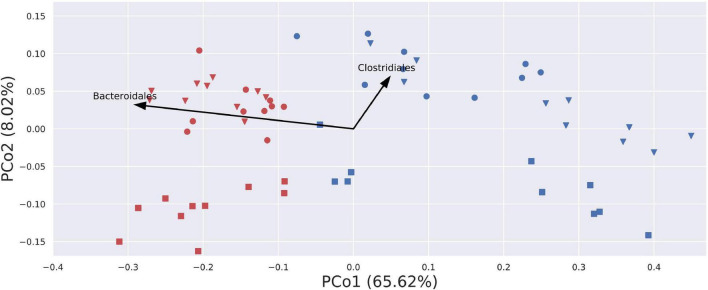
Visualization of PCoA analysis on weighted UniFrac index. Samples are stretched along the x-axis, with a clear division for fresh (red) and blue (frozen) samples. Arrows describe a general trend in the relative abundance of given bacterial order. Only two arrows were plotted, as arrows for other bacterial orders would not be visible due to their short length. Blue – amplicons prepared from frozen stool; red – amplicons prepared from fresh stool; circle – donor A; square – donor B; triangle – donor C.

Beta-diversity analyses using PCoA visualizations on Bray–Curtis (quantitative and qualitative) index and Jaccard (only qualitative) index showed clear, donor-wise clusters, with fresh and frozen samples clustering by the donor. This shows that although freezing introduced some substantial differences in community composition, those differences did not overcome intrinsic inter-donor characteristics. Interestingly, PCoA visualization built using a weighted UniFrac (phylogenetic and qualitative) index showed no donor-wise clusters, but a clear split between fresh and frozen samples (Figure 8). This split can be in part attributed to the changes in the relative abundance of Bacteroidales and Clostridiales orders. While Bacteroidales are more abundant in fresh samples, Clostridiales appear to be more abundant in some of donor A and C frozen samples, as donor B frozen samples are placed mainly in the lower right part of the diagram, and thus being uninfluenced or even negatively influenced by this trend.

**FIGURE 9:**
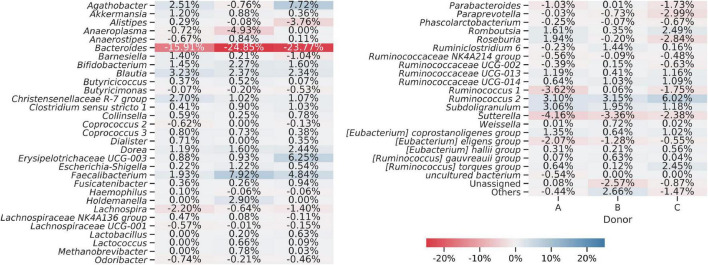
Heat map showing differences in bacterial genera detected from amplicons prepared from the fresh and frozen stool. Averaged relative abundances from fresh samples were subtracted from results obtained for frozen samples, so negative values show that a certain genus was more abundant in fresh samples and positive values show that a genus was more abundant in frozen samples. The “Others” group summarizes genera with individual abundances lower than 0.5% in every sample. Sequences unassigned at the genus level were grouped and named “Unassigned.”

Sequences obtained from fresh and frozen samples were classified using the same methodology, with Silva 132 as a reference database. Figure 9 shows mean and per donor differences in relative abundances between fresh and frozen amplicons at the genus taxonomic level. *Bacteroides* genus was the main source of differences between investigated groups. We observed that *Bacteroides* were more abundant in fresh than frozen samples by an average of 21.51 percentage points. Members of Clostridiales class, which was shown to be characteristic of some of the frozen samples, were much more dispersed, with more than 50 genera contributing to this trend. The biggest contributors to this trend were *Faecalibacterium* (more abundant in frozen samples by 4.89 percentage points), *Ruminococcus 2* group (more abundant in frozen samples by 4.09 percentage points), and *Agathobacter* (more abundant in frozen samples by 3.16 percentage points). However, this trend was not uniform for all donors, as in the case of donor B, members of *Agathobacter* were slightly, by 0.76 percentage points, more abundant in fresh compared to frozen samples.

To statistically test observed differences, we employed ANCOM analysis. ANCOM analysis highlighted eight bacterial classes to differ between fresh and frozen samples. Members of Bacteroidia, Clostridia, Deltaproteobacteria, and Lentisphaeria were more abundant in fresh samples, while members of Actinobacteria, Bacilli, Coriobacteriia, and Erysipelotrichia were more abundant in frozen samples. This result is exceptionally interesting when interpreted with results obtained from a culture-based approach. Culturing showed that the number of bacterial species from Bacilli and Actinobacteria classes was considerably lower in frozen than fresh samples, while NGS analysis showed that their relative abundances were higher in frozen samples. Furthermore, culturing showed similar numbers of species of Bacteroidia class in fresh and frozen samples, while ANCOM analysis showed that Bacteroidia are significantly more abundant in fresh samples. This could mean that in the first case, whole stool freezing, in general, lowers species diversity in Bacilli and Actinobacteria classes. In the second case, we observed a rapid drop in the relative abundance of Bacteroidia, but their species diversity stayed at the same level.

Interestingly, ANCOM analysis on genus level highlighted only two genera—*Sutterella* and *[Eubacterium] eligens* group (member of Lachnospiraceae family), both significantly more abundant in fresh than frozen samples. Although these two taxa constituted no more than 8% of a given sample, due to freezing their relative abundances dropped by the order of 100, and in some samples, they became undetectable.

ANCOM comparisons between fresh and frozen samples from the same donor also showed *Suterella* to be significantly more abundant in fresh samples in each pair. In addition, donors A and B showed two more and donor C one more statistically significant difference at the genus level. In donor A, an “uncultured” genus of Eggerthellaceae family was more abundant in frozen samples. Moreover, ANCOM analysis of donor A also highlighted *Lachnospiraceae ND3007* group to be more abundant in fresh than frozen samples. In donor B, the analysis showed *Anaeroplasma* and an “uncultured” member of *Puniceicoccaceae* family to be more abundant in fresh compared to frozen samples, while comparisons on donor C amplicons highlighted *Lachnoclostridium* genus to be more abundant in fresh samples.

Correlation analysis detected several possible links between NGS and flow cytometry results, for suspensions prepared from both fresh and frozen stools (Supplementary Table 2). Our results show that the biggest number of correlations can be observed for donor B samples and the SYTO9^–^, PI^–^ flow cytometry subfraction. *Anaeroplasma* genus was positively correlated (correlation = 0.9377) with SYTO9^–^, PI^–^ subfraction, and *Holdemanella* was negatively correlated (correlation = –0.6982) with the same subfraction in donor B fresh samples.

## Discussion

Recent reports show that FMT interventions using frozen material deliver similar results to interventions with fresh material only (Burz et al., 2019). In the course of this work, we decided to further elucidate the impact of whole stool freezing on the FMT-ready suspensions microbial community. Our results clearly show that whole stool freezing without any cryopreserving buffer significantly alters stool microbiota. This change can be detected using either of the methods we employed, that is, flow cytometry with cell staining, classical culturing, and metabarcoding.

Flow cytometry with cell staining showed that freezing causes significant changes in all of the observed fractions. Alive cell counts dropped four times, from around 70% to 15%, while the other two fractions, dead and unknown, cell counts quadrupled and doubled, with the Unknown fraction becoming the dominant one, with an average contribution of 57.47% per sample. These changes were observed for all three donors and we did not detect any significant differences between them. However, it is important to notice that if we were to apply flow cytometry without cell staining (discriminating dead and alive cells), we would observe no differences between fresh and frozen samples. This observation additionally highlights the fact that here NGS results may be significantly biased as being based mainly on DNA from the dead cells, and therefore the differences in actual alive, active community composition may be much bigger if cell sorting for alive and dead fraction was used before DNA isolation. Nevertheless, FMT with frozen material still appears to deliver satisfactory results, as the whole stool freezing approach is routinely used in one of the polish stool banks with an overall CDI cure rate approx. 90% (Grzesiowski et al., 2015). We also consider it very important to note that the lack of characterization of cells from the Unknown group, and especially from the group of “double negative” cells, does not allow us to clearly state whether the viability of bacterial cells has actually decreased, whether it has not changed or even increased during freezing. This has not been the subject of this experiment. However, the next step must be to characterize this population after FACS cell sorting and then plating to see if these cells start to proliferate and what species they are. It may turn out that these “double negative cells” are simply bacterial spores that will start to proliferate when they are returned to favorable incubation conditions, which would mean that, after freezing, the viability of the preparations could even increase. It may also be a shift toward cells with a thick cell wall, which may be suggested by the fact that the Unknown fraction correlates, for example, with bacteria of the *Anaeroplasma* genus, known as bacteria with a thick cell wall.

Classical culturing showed a significant drop in the number of detected bacterial species as a result of freezing. Among four detected bacterial phyla, Bacteroidetes and Bacilli (of Firmicutes) showed the highest decrease in the number of observed cultivable bacterial species (Figure 7). As in the case of flow cytometry, the changes were similar between all donors; however, donor C (the regular stool donor) showed the highest drop in the number of unique cultivable species, with 63 species detected in fresh and only 31 in frozen samples. Applied sampling methodology, 10 stools from each donor over 10 days, also allowed us to notice a very high variability in the number of detected bacterial species. That led to the conclusion that time-resolved sampling is crucial to obtain replicable results if only culturing is employed for stool quality assessment. It is also very interesting when combining culture data with flow cytometry and NGS. The biggest drop of culturable cells in donor C, with the information of mostly sporulating bacteria as microbiota components in this donor and a significant increase in the “unknown” fraction in flow cytometry, may lead us to the conclusion that freezing may induce massive spore-forming in those sporulating bacteria. However, this needs further investigation.

Metabarcoding was the method that delivered the most comprehensive results; however, it must be noted that we did not perform any cell fractioning before DNA isolation, and as a result, all DNA available in the samples, both from alive and dead cells, was jointly sequenced. Inspection of basic alpha-diversity indices showed no significant differences between fresh and frozen samples from most donors. We observed a decrease in the relative abundance of bacteria from Bacteroidales order, while the *Faecalibacterium* genus relative abundances increased in FMT suspensions after freezing. PCoA visualization of weighted UniFrac distance metric showed clear separation of fresh and frozen samples, with Bacteroidales being more abundant in fresh than frozen samples (Figure 8). ANCOM analysis of NGS results highlighted several taxa with statistically significant differences between investigated groups. Members of Bacteroidia, Clostridia, Deltaproteobacteria, and Lentisphaeria were more abundant in fresh samples, while members of Actinobacteria, Bacilli, Coriobacteriia, and Erysipelotrichia were more abundant in frozen samples. Surprisingly, ANCOM analysis on genus level highlighted only two genera—*Sutterella* and *[Eubacterium] eligens* group (member of Lachnospiraceae family), both significantly more abundant in fresh than frozen samples.

We also investigated the possibility that whole stool freezing without any pre-processing could promote the preservation of anaerobic bacteria. Culturing experiments showed no statistically significant differences in this matter. We were not able to validate this hypothesis on NGS data, as there are no databases that would allow us to properly classify obtained ASVs as either aerobic or anaerobic.

In addition, we hypothesized that the obtained results would allow us to highlight some of the key characteristics of “a good donor.” We based the correlations with donor C samples (regular stool donor). Interestingly, freezing had the biggest impact on donor C samples. Classical cultivation experiments showed the biggest decrease in the number of cultivable bacterial species in this case, as 5 out of 12 bacterial classes identified in fresh samples were undetectable after freezing. This decrease in diversity is also visible upon inspection of Shannon’s Diversity and Faith’s Phylogenetic Diversity indices. While donor C’s fresh samples showed the highest mean values in these indices, biodiversity in suspensions prepared from the frozen stool samples of donor C was lower than for other donors. Cultivation experiments showed three bacterial species unique only to donor C, which were *Bacteroides cellulosilyticus*, *Enterococcus avium*, and *Enterococcus thailandicus*. All three are common human gut microbiota, with the first one characterized by its versatile metabolic potential, especially in carbohydrate hydrolysis, and its high activity in heparinase I (Ali-Ahmad et al., 2017). *Enterococcus avium*, while more often found in birds than human microflora, is considered a human pathogen due to its wide array of virulence factors (Yu et al., 2019). Not much is known about *Enterococcus thailandicus*, but its presence only in donor C is exceptionally interesting, as a recent report by Li et al. (2021) described *E. thailandicus* d5b strain with very potent antimicrobial activity against *C. difficile* (Li et al., 2021). These final observations raise several novel questions that will be used as a foundation for our further research.

## Data Availability Statement

The original contributions presented in this study are publicly available. This data can be found here: http://www.ebi.ac.uk/, PRJEB36368.

## Ethics Statement

Ethical review and approval was not required for the study on human participants in accordance with the local legislation and institutional requirements. The patients/participants provided their written informed consent to participate in this study.

## Author Contributions

JB, MD, ŁD, and GB designed the study, performed all analyses, and wrote the article. JB, MD, PG, EP, AS-E, TD, KL, MT, and KO performed the experiments and commented on the previous version of the manuscript. All authors contributed to the article and approved the submitted version.

## Conflict of Interest

The authors declare that the research was conducted in the absence of any commercial or financial relationships that could be construed as a potential conflict of interest.

## Publisher’s Note

All claims expressed in this article are solely those of the authors and do not necessarily represent those of their affiliated organizations, or those of the publisher, the editors and the reviewers. Any product that may be evaluated in this article, or claim that may be made by its manufacturer, is not guaranteed or endorsed by the publisher.
