# Supplementary material for: Fresh Versus Frozen Stool for Fecal Microbiota Transplantation—Assessment by Multimethod Approach Combining Culturing, Flow Cytometry, and Next-Generation Sequencing
Source: Front Microbiol. 2022 Jul 1;13:872735. doi: 10.3389/fmicb.2022.872735 (PMC9284506; doi:10.3389/fmicb.2022.872735)
Supplement: Supplementary file 1 [file Data_Sheet_1.zip › Data sheet 1/Figure S1.docx]

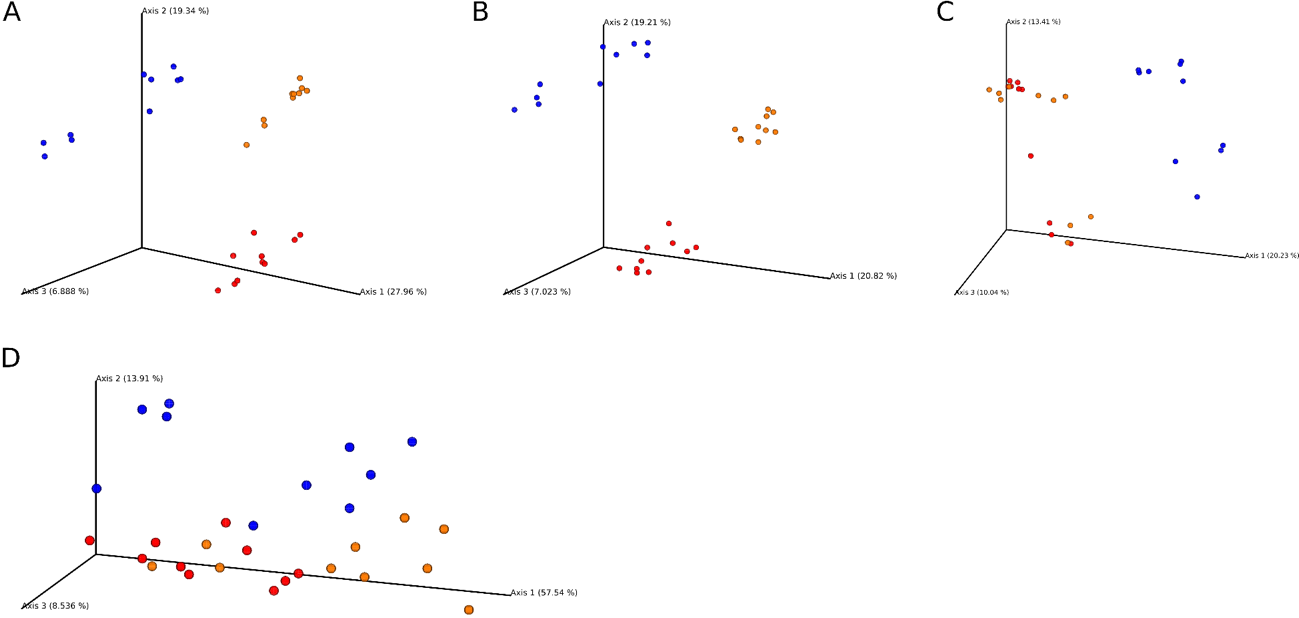


Figure S1. PCoA visualization of selected beta-diversity indices for frozen samples. A: PCoA of Bray-Curtis Dissimilarity index; B: PCoA of Jaccard index; C: PCoA of Unweighted Unifrac index; D: PCoA of Weighted Unifrac index; blue dots: donor A; red dots: donor B; orange dots: donor C.
