# Supplementary material for: Fresh Versus Frozen Stool for Fecal Microbiota Transplantation—Assessment by Multimethod Approach Combining Culturing, Flow Cytometry, and Next-Generation Sequencing
Source: Front Microbiol. 2022 Jul 1;13:872735. doi: 10.3389/fmicb.2022.872735 (PMC9284506; doi:10.3389/fmicb.2022.872735)
Supplement: Supplementary file 1 [file Data_Sheet_1.zip › Data sheet 1/Table S1 and S2 captions.docx]

Table S1. Summary of raw reads quality control with filtering, trimming, denoising and chimera removal using Dada2 library in QIIME2 environment.

Table S2. Table showing results of cross-correlation analysis between cell counts obtained from flow cytometry experiment and relative abundance from the NGS experiment. Each sheet refers to one taxonomic level. Only statistically significant (p-value < 0.05) are presented.
